# Multipocket synergy towards high thermoelectric performance in topological semimetal TaAs_2_

**DOI:** 10.1038/s41467-024-55490-6

**Published:** 2025-01-02

**Authors:** Haihua Hu, Xiaolong Feng, Yu Pan, Vicky Hasse, Honghui Wang, Bin He, Claudia Felser

**Affiliations:** 1 Max Planck Institute for Chemical Physics of Solids, Nöthnitzer Str. 40, Dresden, Germany; 2 College of Materials Science and Engineering and Center of Quantum Materials & Devices, Chongqing University, Chongqing, 400044 China

**Keywords:** Thermoelectrics, Topological matter

## Abstract

Charge-carrier compensation in topological semimetals amplifies the Nernst signal and simultaneously degrades the Seebeck coefficient. In this study, we report the simultaneous achievement of both a large Nernst signal and an unsaturating magneto-Seebeck coefficient in a topological nodal-line semimetal TaAs_2_ single crystal. The unique dual-high transverse and longitudinal thermopowers are attributed to multipocket synergy effects: the combination of a strong phonon-drag effect and the two overlapping highly dispersive conduction and valence bands with electron–hole compensation and high mobility, promising a large Nernst effect; the third Dirac band causes a large magneto-Seebeck effect. High transverse and longitudinal power factors of ~3100 and ~50 μW cm^−1^ K^−2^, respectively, are achieved, surpassing those of other topological semimetals and mainstream semiconductors. Our study presents a feasible approach for optimizing the longitudinal and transverse thermopowers in topological semimetals simultaneously and demonstrates the potential of TaAs_2_ for low temperature solid-state cooling.

## Abstract (web summary)

The electron-hole compensation hinders the magneto-Seebeck coefficient of topological semimetals. Here, authors report simultaneous high longitudinal and transverse thermopowers in a TaAs_2_ single crystal by a multipocket synergy strategy.

## Introduction

Explorations of the intriguing transport properties and quantum behaviors of topological semimetals have enriched the scope of topological quantum materials for advanced energy conversion technologies, such as spin-caloritronics, topological catalysis, and thermoelectrics^1–5^. Thermoelectric technology, which realizes the direct conversion between heat and electricity, has significant potential for power generation from waste heat and electronic refrigeration via solid-state cooling^6–8^. The output power of a thermoelectric device is determined by the power factor^9^, defined as *PF* = *S*^2^/*ρ*, where *S* and *ρ* represent the thermopower and resistivity, respectively. Depending on the configuration of the output voltage and applied temperature gradient, there exists a longitudinal Seebeck effect and a transverse Nernst effect, where the output voltage is along the same direction as the temperature gradient in the Seebeck effect, whereas the output voltage is perpendicular to the temperature gradient in the Nernst effect under magnetic fields. Therefore, the Seebeck and Nernst power factors are defined as *PF*_S_ = *S*_xx_^2^/*ρ*_xx_ and *PF*_N_ = *S*_yx_^2^/*ρ*_yy_, respectively, where *S*_xx_ and *S*_yx_ represent Seebeck and Nernst thermopower, respectively. In recent years, topological semimetals have attracted significant attention for the Nernst effect because of their exceptionally high performance^10–15^. Further, the Nernst device fabrication process is significantly simplified because the indispensable paired assemblies encountered in the Seebeck device are unnecessary, which helps to reduce the electrical and thermal resistance in the modules^16,17^.

A more efficient and straightforward cooling system capable of multi-directional refrigeration can be achieved by combining transverse Nernst and longitudinal Seebeck thermopowers^18^. A high *S*_yx_ is expected in a two-carrier system with high mobility and electron–hole compensation because electrons and holes are deflected in opposite directions by the magnetic field in the Nernst effect. However, the two-carrier transport behavior is detrimental to the Seebeck coefficient because the joint transport of electrons and holes in the same direction reduces the accumulated Seebeck voltage. Consequently, it is considerably challenging to achieve high Nernst thermopower and Seebeck coefficients simultaneously in a single material. Solving this dilemma relies on introducing electrons and holes with different mobilities in different pockets. For example, in Dirac or Weyl semimetals with high mobilities, a large magneto-Seebeck effect can be experimentally expected when the topological quantum effect is introduced because of the linear band dispersion *E* = ℏ*kv*^19,20^. Meanwhile, electrons and holes in other pockets that have lower mobilities can contribute to the Nernst effect.

**Fig. 1:**
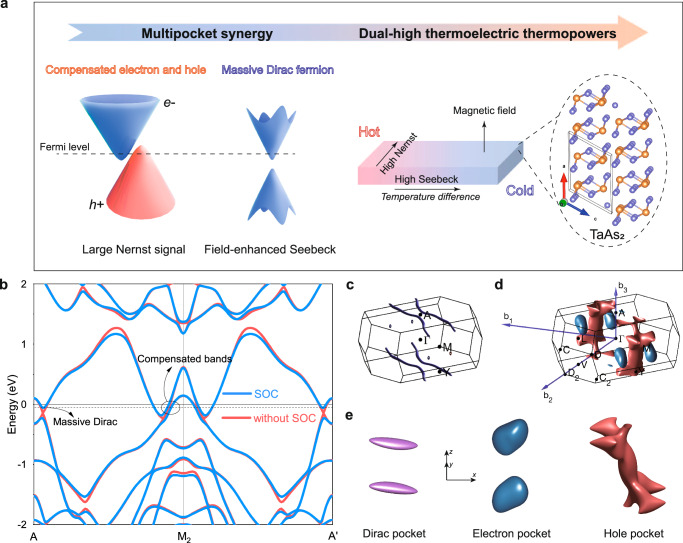
Multipocket synergy leading to high thermopowers. **a** Schematic of multipocket synergy in the TaAs_2_ crystal that boost the high-thermoelectric thermopower. The pockets with electron–hole compensation contribute to a large Nernst signal, and a massive Dirac pocket enhances the Seebeck coefficient under an applied field. **b** Electronic band structures of TaAs_2_ with and without spin-orbit coupling. Band crossings along the M-A direction are presented without the SOC. The dashed line represents the actual Fermi level. **c** Calculated nodal lines in the first Brillouin zone. **d**, **e** Fermi surfaces of two bands with spin-orbit coupling calculations. Fermi pockets in (**e**) correspond to the massive Dirac pocket (purple), compensated electron pocket (blue), and compensated hole pocket (red).

In this work, we simultaneously achieved large longitudinal and transverse thermopowers in a nodal-line topological semimetal TaAs_2_ single crystal using a multipocket synergy strategy (Fig. 1a). Since the topological semimetal TaAs_2_ has a centrosymmetric monoclinic structure with a space group of *C*2/m (no. 12), the Berry curvature is zero and the anomalous Nernst effect is not considered, even under magnetic since the external magnetic field is insufficient to induce a decent non-zero Berry curvature in TaAs_2_, which is different from Cd_3_As_2_ observed by previous studies^21,22^. Theoretical calculations were conducted to investigate the effect of multipocket synergy on the dual-high thermoelectric thermopowers of TaAs_2_. The electronic band structures of TaAs_2_ without and with spin-orbit coupling (SOC) are shown in Fig. 1b and Supplementary Fig. 1. Without the SOC, the anti-crossing band near points A and M formed nodal lines. These nodal lines can be classified into two types within the Brillouin zones: one type includes two open spiral nodal lines that extend across the Brillouin zones through point A, while the second type comprises two closed nodal loops near the M point (Fig. 1c). After accounting for the SOC effect, the nodal lines gap out the anticrossing feature^23,24^, resulting in a massive Dirac fermion that aligns with the zero-field massive Dirac dispersion relation^19^

1 $${{{E}}}_{{{{\rm{Dirac}}}}}=\sqrt{{\left(\frac{\Delta }{2}\right)}^{2}+{\hslash }^{2}{\upsilon }_{{{{\rm{F}}}}}^{2}{{{{\rm{K}}}}}^{2}}$$

where ∆ and *ν*_F_ represent the energy gap and Fermi velocity, respectively (Supplementary Fig. 1c–e). Therefore, there are three Fermi pockets crossing the Fermi level *E*_F_, where the hole pocket centered at the M point and electron pocket close to the M point can result in an electron–hole compensation behavior, and the massive Dirac pockets around point A may produce a large magneto-Seebeck effect because of the quantum effect^19,20^. Figure 1d shows the three types of Fermi surfaces present in the first Brillouin zone of TaAs_2_. The hole pocket is located at the M point, while the large electron pocket can be found close to the M point, and there is also a small electron pocket near point A. The mapped Fermi surfaces (Fig. 1e) reveal that the electron (blue) and hole (red) pockets possess similar volumes, indicating a nearly perfect compensation of electrons and holes near the Fermi level.

## Results

### Electrical transport properties

**Fig. 2:**
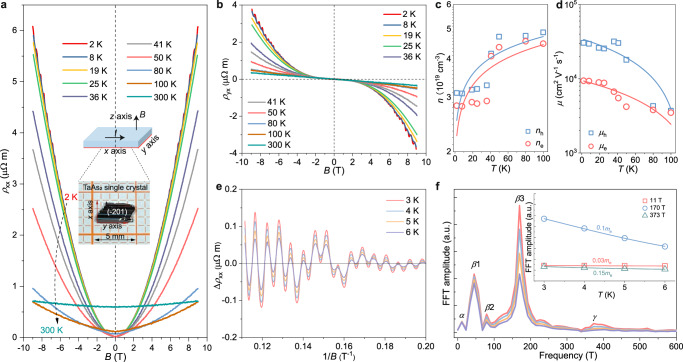
Electrical transport properties and quantum oscillations. **a** Magnetic field dependence of longitudinal resistivity *ρ*_xx_. The inset displays a photograph of the TaAs_2_ single crystal shaped like a bar with a length of ~5 mm and a width of 3 mm. The ($\bar{2}$01) crystal plane is the largest exposed surface of the TaAs_2_ single crystal. The schematic indicates the electrical current along the *x* axis and magnetic field along the *z* axis. **b** Hall resistivity *ρ*_yx_ as a function of the magnetic field. **c** Carrier concentration. **d** Carrier mobility. **e** Amplitudes of resistivity oscillations as a function of 1/*B* obtained by subtracting a continuous polynomial. **f** Fast Fourier transform (FFT) spectra at different temperatures showing peaks corresponding to holes and electron pockets. The frequencies *α*, *β*, and *γ* represent the massive Dirac pocket, compensated hole pocket, and compensated electron pocket, respectively. The inset displays the effective mass calculations of different Fermi pockets from the Lifshitz–Kosevich formula.

High-quality TaAs_2_ single crystals (Supplementary Figs. 2, 3, and Fig. 2a) were used to investigate the transport properties. The TaAs_2_ single crystal exhibited a distinguishable positive magnetoresistance (Fig. 2a, Supplementary Fig. 4) without reaching saturation up to 9 T at low temperatures, suggesting typical semimetal behavior. The distinct nonlinear field dependence of the Hall resistivity (Fig. 2b) indicates the collaborative contribution of both hole and electron carriers to electrical transport, which is consistent with the band structure calculations. Furthermore, carrier concentration and mobility were determined using a semiclassical two-carrier model based on longitudinal and Hall resistivity measurements^25^. The fitting formulae and results are detailed in Supplementary Fig. 5, and the carrier parameters are presented in Fig. 2c, d. The concentrations of electrons (*n*_e_) and holes (*n*_h_) are comparable over the entire temperature range, indicating a near compensation of holes and electrons. For example, *n*_e_ and *n*_h_ are ~2.8 × 10^19^ cm^−3^ and ~3.1 × 10^19^ cm^−3^ at 2 K, respectively. When the temperature ranges from 2 to 100 K, *n*_e_ and *n*_h_ exhibit a similar increase. The compensation of charge carriers and relatively low carrier concentrations in the TaAs_2_ crystals provide additional evidence for their semi-metal nature. Besides the carrier compensation, high charge carrier mobilities also play a critical role in determining the large Nernst thermopower^12^. The hole mobility (*µ*_h_) of the TaAs_2_ crystal is ~3.1 × 10^4^ cm^2^ V^−1^ s^−1^ at 2 K, whereas the electron mobility (*µ*_e_) is ~0.94 × 10^4^ cm^2^ V^−1^ s^−1^. These values validate the superior quality of the TaAs_2_ crystal and can be compared to those of topological semimetals that have been previously reported, such as NbSb_2_ (*µ*_e_ = 2.1 × 10^4^ cm^2 ^V^−1^ s^−1^ and *µ*_h_ = 1.2 × 10^4^ cm^2^ V^−1^ s^−1^ at 5 K)^10^, and NbAs_2_ (*µ*_e_ = 3.6 × 10^4^ cm^2^ V^−1^ s^−1^ and *µ*_h_ = 7.2 × 10^4^ cm^2 ^V^−1^ s^−1^ at 2 K)^13^.

Further, both *ρ*_xx_ and *ρ*_yx_ exhibit distinct quantum Shubnikov–de Haas (SdH) oscillations at magnetic fields above 5 T below 8 K, helping analyze the shape of Fermi surfaces^26–28^. The variation trend of the SdH oscillation amplitudes with an inverse magnetic field is shown in Fig. 2e, demonstrating their sensitivity to temperature differences. The amplitude of the longitudinal resistivity oscillations progressively weakened with increasing temperature. A fast Fourier transform (FFT) analysis of the oscillatory part revealed that there are five fundamental frequencies at *F* = 11 T (*α*), 45 T (*β*1), 79 T (*β*2), 179 T (*β*3), and 373 T (*γ*) (Fig. 2f), which are in good agreement with those reported in the previous works^29,30^. Frequencies *α*, *β*, and *γ* are assigned to the massive Dirac pocket, compensated hole pocket, and compensated electron pocket, respectively. We can determine the effective masses m* (inset of Fig. 2f) of the three Fermi pockets using the Lifshitz–Kosevich formula by analyzing the temperature dependence of the SdH amplitudes *A*_FFT_: *A*_FFT_ = *A*_0_*G*/sinh (*G*), where *A*_0_ represents a constant, *G* = 14.69 m*T/*B*, 1/*B* = 1/*B*_1_ + 1/*B*_2_, and *B*_1_ and *B*_2_ represent the initial and final magnetic fields of the FFT field window^26^. According to the fitting results, the effective masses of the three Fermi pockets are 0.03*m*_e_ (*α*), 0.1*m*_e_ (*β*3), and 0.15*m*_e_ (*γ*), with *m*_e_ representing the free electron mass. Therefore, the high carrier mobility of the TaAs_2_ crystal can be attributed to its small effective mass and linear dispersion bands.

### Thermal transport properties

**Fig. 3:**
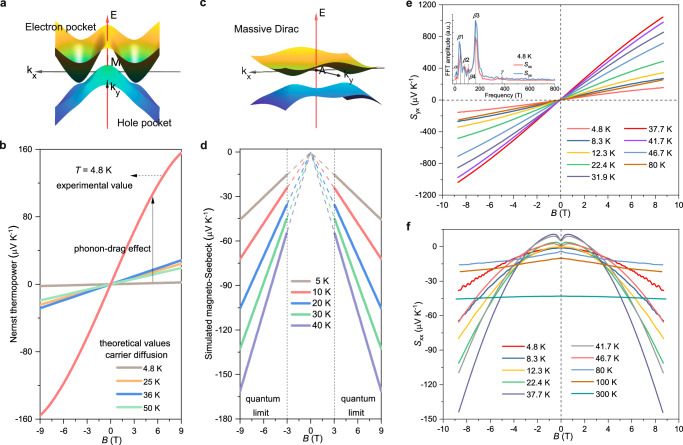
Thermoelectric thermopowers. **a** Schematic of the compensated electron–hole pockets near the Fermi energy. **b** Field-dependent Nernst thermopower related to the charge carrier diffusion processes (calculation) and phonon-drag effect (experiment). **c** Spin-orbit coupling gapped the nodal lines into massive Dirac pockets close to point A. **d** The magnetic field-dependent Seebeck coefficient of the massive Dirac pocket in TaAs_2_ based on the theoretical model in the extreme quantum limit ( ± 3 ~ ±9 T). Magnetic field dependence of (**e**) Nernst thermopower *S*_yx_, and **f** Seebeck coefficient *S*_xx_ at different temperatures. The inset shows the FFT spectra of *S*_xx_ and *S*_yx_ at 4.8 K.

Compensated high mobility electrons and holes near the Fermi level, along with a strong phonon-drag effect, can lead to exceptional transverse thermoelectric performance. Figure 3a shows the compensated electron–hole pockets we focused on for calculating the Nernst thermopower. The Nernst thermopower related to the charge carriers diffusion processes at different temperatures was calculated based on the experimental mobilities, carrier concentrations, and effective masses (Fig. 3b). Further information on how this calculation was carried out is provided in Supplementary Note 1. The experimental Nernst thermopower reveal a maximum value of 156 μV K^−1^ at 4.8 K and 9 T, significantly higher than the thermopower related to the charge carriers diffusion processes (~2 μV K^−1^) at the same temperature and magnetic field. The exceptional large Nernst thermopower at low temperatures is usually attributed to the phonon-drag effect^10^. The experimental Nernst thermopower *S*_yx_ of the TaAs_2_ single crystal as a function of the magnetic field is shown in Fig. 3e. *S*_yx_ is nonlinearly corrected with unsaturated values with a magnetic field ranging from 4.8 to 46.7 K; however, it starts to deviate from this pattern gradually once the temperature exceeds 46.7 K. The transition from a nonlinear to linear Nernst signal in TaAs_2_ single crystal indicates a slight discrepancy in carrier compensation at temperatures below 46.7 K, while an almost perfect compensation can be obtained once the temperature surpasses this threshold. With increasing temperature, the *S*_yx_ value rises under a magnetic field of 9 T, reaching a peak of ~1045 µV K^−1^ at 37.7 K before decreasing at higher temperatures. Moreover, a two-carrier model was used to analyze the behavior of the Nernst thermopower in TaAs_2_. The Seebeck coefficients for electrons (*S*_xx_^e^) and holes (*S*_xx_^h^) deviate from linear temperature dependence below 100 K and exhibit peaks around 45 K, indicating a significant contribution from phonons (*S*_p_) to the Seebeck coefficient at low temperatures. The absolute values of *S*_p_^e^ and *S*_p_^h^ reach maximum values of 73 μV K^−1^ and 76 μV K^−1^ around 45 K, which are much larger than the values of *S*_d_^e^ (4.1 μV K^−1^) and *S*_d_^h^ (2.6 μV K^−1^) at the same temperature. This suggests that the phonon-drag effect significantly enhances the total Nernst effect in single-crystalline TaAs_2_^10,31^. Additional details can be found in Supplementary Note 1 and Supplementary Fig. 6. Additionally, the thermal conductivity behavior (Supplementary Fig. 7) can be used as further evidence to support the phonon-drag effect.

Unlike the Nernst signal, understanding the magneto-Seebeck effect of a massive Dirac pocket (Fig. 3c) requires the involvement of a topological quantum picture. The thermopower of a Dirac semimetal undergoes quantum oscillations when an increasing magnetic field is applied because of the depopulation of higher Landau levels. Once the magnetic field reaches a certain strength where ℏ*v*_F_/*l*_B_ > *E*_F_ (*l*_B_ = $\sqrt{\hslash /{eB}}$ represents the magnetic length), the system crosses into the extreme quantum limit, resulting in a notable magnetic field-induced variation in the Fermi energy (µ $\propto$ 1/*B*) and density of states (*D*(µ) $\propto$ *B*)^20^. In the extreme quantum limit, the Seebeck coefficient can be expressed as ${S}_{{{{\rm{xx}}}}}\simeq \frac{{\kappa }_{{{{\rm{B}}}}}^{2}{NTB}}{6{\hslash }^{2}{\upsilon_{{{\rm{F}}}}}{C}}$, where *κ*_B_, *N*, *T*, and *C* represent the Boltzmann constant, number of Dirac nodes, temperature, and carrier concentration, respectively. As the density of states increases, *S*_xx_ also exhibits a linear non-saturated increase with the magnetic field, leading to a large Seebeck coefficient. More quantitatively, the theoretical simulation results in Fig. 3d show a linear correlation between the Seebeck coefficient of TaAs_2_ and the magnetic field, confirming that the large Seebeck coefficient originates from a massive Dirac fermion.

We measured the magnetic-field-dependent Seebeck coefficient *S*_xx_ to investigate the influence of the massive Dirac fermion on the longitudinal thermoelectric performance, as depicted in Fig. 3f. The reversal of the *S*_xx_ sign at various temperatures or magnetic fields is linked to the transition of the carrier type between electrons and holes, potentially caused by the slight shift in the Fermi level, which results in asymmetric conduction and valence bands^32,33^. A remarkably low *S*_xx_ value of less than 15 μV K^−1^ in the absolute value is obtained when the temperature is below 100 K and the magnetic field is less than 2.5 T. With the magnetic field exceeding 2.5 T, the *S*_xx_ data demonstrates a more noticeable upward trend as the magnetic fields intensify across the temperature range, particularly exhibiting an unsaturated super linear increase between 22.4 K and 41.7 K. Under 9 T and 37.7 K, the maximum *S*_xx_ reaches about −144 μV K^−1^, ~27 times the value at 0 T. The unsaturated magneto-Seebeck effect is caused by the massive Dirac pocket entering the extreme quantum limit, resulting a linear increase. Moreover, evident quantum oscillations are detected in both the Nernst thermopower and Seebeck coefficient below 8.3 K. The FFT analysis of the *S*_xx_ and *S*_yx_ oscillations at 4.8 K reveals five main frequencies, which is in accordance with the magnetoresistance results, as depicted in the inset of Fig. 3e and Supplementary Fig. 6. Moreover, an additional frequency *β*4 (106 T) is observed, possibly related to an irregular hole pocket as reported previously. The thermal transport oscillation, being the energy derivative, is more sensitive than the SdH, explaining why this frequency is not observed in the SdH analysis^29,34^.

### Thermoelectric power factors

**Fig. 4:**
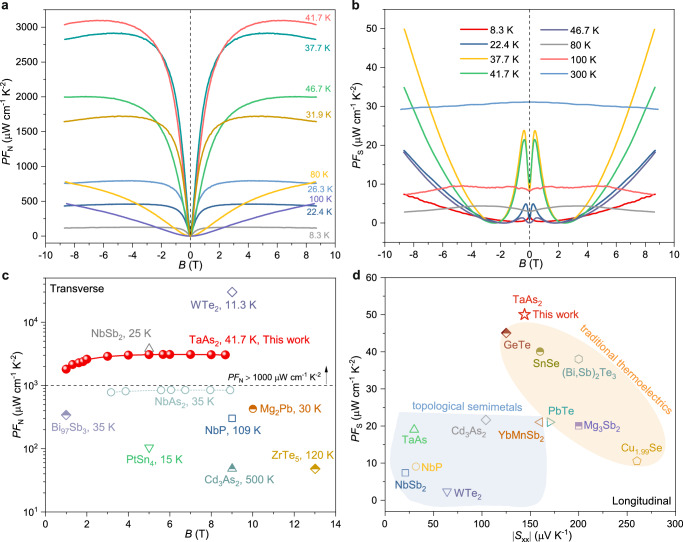
Power factors. Magnetic field dependence of (**a)** transverse power factor *PF*_N_ and (**b)** longitudinal power factor *PF*_S_ at different temperatures. Comparison of the (**c**) Nernst power factor and **d** longitudinal power factor of TaAs_2_ with those of reported topological semimetals and thermoelectric semiconductors^10–13,18,35–37,41,48–56^. In order to eliminate the influence of other mechanisms, all the data are taken from single crystals and polycrystals without any further doping or alloying.

In addition to the large Nernst thermopower and Seebeck coefficient, the transverse and longitudinal power factors are key for determining the heat-pumping power and output power density of thermoelectric devices. Figure 4a, b show the magnetic-field-dependent transverse and longitudinal power factors, respectively. At temperatures below 80 K, the *PF*_N_ showed a rapid increase in low fields, eventually leveling off at a saturated value. This results in a peak value of ~3100 μW cm^−1^ K^−2^ at 9 T and 41.7 K, ranking at the top level among topological materials (Fig. 4c). An exceptional high *PF*_N_ (exceeding 460 μW cm^−1^ K^−2^) can be retained even at a high temperature of 100 K. When the temperature falls below 46.7 K and the magnetic field goes above 2.5 T, the *PF*_S_ of the TaAs_2_ crystal exhibits a sharp rise, peaking at 50 μW cm^−1^ K^−2^ at 37.7 K and 9 T. Even at 300 K, the *PF*_S_ remains at 31 μW cm^−1^ K^−2^ at 0 T, comparable to that of the state-of-the-art thermoelectric materials such as PbTe (~21 μW cm^−1^ K^−2^)^35^, Mg_3_Sb_2_ (~20 μW cm^−1^ K^−2^)^36^, and Cu_1.99_Se (~10.5 μW cm^−1^ K^−2^)^37^ (Fig. 4d). Moreover, similar to the case for most topological semimetal single crystals, TaAs_2_ demonstrates high thermal conductivity *κ* (see Supplementary Fig. 7). Considering the large power factors and high thermal conductivity, we can obtain transverse (*Z*_N_, *Z*_N_*T*, defined as *Z*_N_ = *PF*_N_/*κ*) and longitudinal figure of merits (*Z*_S_, *Z*_S_*T*, defined as *Z*_S_ = *PF*_S_/*κ*, Supplementary Fig. 8); their variation trend is similar to that of *PF*_N_ and *PF*_S_. A potential reduction in thermal conductivity can be achieved by reducing the carrier concentration and enhancing phonon scattering in future studies to achieve the high *Z*_N_*T* ^38–40^. Thus, the as-prepared TaAs_2_ single crystal is a promising candidate for thermoelectric cooling because it combines longitudinal and transverse thermoelectric effects.

## Discussion

The coexistence of dual-high magneto-Seebeck coefficients and Nernst thermopowers achieved through a multipocket synergy strategy has rarely been reported. Prior to this study, Fu et al. had already investigated the Nernst power factor in polycrystalline NbP through a multipocket strategy^41^. Nevertheless, the motivation and outcomes of our study diverge significantly from theirs. While they relied on a combination of small and large electron pockets to compensate for a large hole pocket for enhanced Nernst thermopowers and power factors, they did not delve into the significance of the small pocket in the Seebeck coefficient. Additionally, it should be noted that ZrTe_5_ is the only material known to possess large values of both magneto-Seebeck coefficients and Nernst thermopowers concurrently. Unlike ZrTe_5_, which is recognized as a single-carrier system, the TaAs_2_ crystal investigated in this study is a multipocket system with a massive Dirac pocket that contributes to its remarkable non-saturating Seebeck coefficient. The absence of inter-valley scattering, which normally balances the carrier concentrations between different pockets and decreases both the Seebeck and Nernst thermopowers, can be attributed to the low temperature, where the phonons responsible for scattering are not yet active, preventing interactions between the electrons in each pocket. However, as the temperature increased, the phonon wave vector also increased. When the phonon wave vector approached the size of the Brillouin zone, strong scattering occurred, causing the two pockets to merge and decrease both *S*_xx_ and *S*_yx_. In addition, we discovered that the thermal conductivity reached its peak at 37.7 K, which was similar to the peaks of *S*_xx_ and *S*_yx_. Below 40 K, the mobilities of both electrons and holes remained relatively constant; however, above this temperature, the mobility decreased rapidly, leading to a decrease in *S*_yx_.

The multipocket synergy strategy was successfully employed to optimize both the longitudinal and transverse thermopowers in the topological semimetal TaAs_2_ single crystal, yielding a high Nernst power factor of approximately 3100 μW cm^−1^ K^−2^ and a high Seebeck power factor of ~50 μW cm^−1^ K^−2^. This strategy provides opportunities for improving the thermoelectric performance of topological semimetals, which represents a significant advancement in thermoelectric performance modulation. Hence, other two-carrier topological semimetals, such as NbSb_2_^10^, NbAs_2_^13^, VP_2_, and VAs_2_ (Supplementary Fig. 9), which possess two compensated pockets and an extra massive Dirac pocket, could serve as promising materials for achieving simultaneous enhancement of both the transverse and longitudinal thermoelectric performances by adjusting the Fermi level. Furthermore, topological semimetals exhibit considerable potential in applications relevant to longitudinal Peltier cooling and transverse Ettingshausen cooling because of the multipocket synergy effect. This can result in a more efficient and straightforward thermoelectric cooling system capable of multidirectional refrigeration.

## Methods

### Sample synthesis and characterization

TaAs_2_ single crystals were synthesized using the chemical vapor transport method. Initially, a blend of high-purity tantalum and arsenic powders in a 1:2 molar ratio, were mixed with 0.1 g of iodine as the transport agent. The mixture was subsequently placed in an evacuated fused silica ampoule and allowed to react at 1073 K for more than 90 h. Subsequently, the transport reaction was conducted in a two-zone furnace with a temperature gradient ranging from 1273 to 1173 K over several weeks. Upon the completion of the reaction, the ampoule was removed from the furnace and rapidly quenched in water. Finally, bar-shaped crystals with shiny surfaces are obtained. Single crystallinity was examined using Laue X-ray diffraction, and the chemical composition was analyzed using scanning electron microscopy (Philips XL30) and Oxford energy-dispersive X-ray spectroscopy (Quantax, Bruker).

### Transport property measurements

The longitudinal and Hall resistivities were measured using a physical property measurement system (PPMS9, Quantum Design) in an electrical transport option with a standard heater and two thermometers. The Nernst thermopower, Seebeck coefficient, and thermal conductivity were also measured using the PPMS9 under high vacuum via a standard four-contact steady-state method. To calibrate any contact misalignments, all collected data were field-symmetrized and antisymmetrized.

### Calculations

First-principles calculations were performed based on the density functional theory implemented in the Vienna ab initio Simulation Package described by the projector augmented wave method^42–45^. The exchange-correlation interaction was included via generalized gradient approximation and parameterized using the Perbew–Burke–Ernzerhof functional^46^. The kinetic energy cut-off was set to 480 eV. A 13 × 13 × 6 *k*-mesh was adopted for Brillouin zone sampling. The energy convergence criterion was set to 10^−6^ eV. Lattice parameters with *a* = 4.963 Å and *c* = 7.752 Å were used in the calculation. To calculate the Fermi surface, a Wannier tight-binding Hamiltonian based on the Ta 5 *d* and As 4*p* orbitals was constructed using the Wannier90 package^47^. In order to determine the quantum oscillation frequencies, the Fermi pockets are examined by slicing them perpendicular to the magnetic field. The frequencies are then calculated from the local extrema using the Onsager relation. By comparing these calculated oscillation frequencies with experimental data, the Fermi level is determined. This is accomplished by testing various Fermi energies, as shown in Supplementary Fig. 1.

## Supplementary information

Supplementary Information

Transparent Peer Review file

## Data Availability

All data necessary to understand and assess this manuscript are shown in the main text and the Supporting Information. The data that support the findings of this study are available from the corresponding author upon reasonable request.
