# Supplementary material for: Multipocket synergy towards high thermoelectric performance in topological semimetal TaAs2
Source: Nat Commun. 2025 Jan 2;16:119. doi: 10.1038/s41467-024-55490-6 (PMC11696835; doi:10.1038/s41467-024-55490-6)
Supplement: Supplementary file 1 — Supplementary Information [file 41467_2024_55490_MOESM1_ESM.pdf]

Supplementary Information

Multipocket synergy towards high thermoelectric performance in topological semimetal $TaAs_2$

Haihua Hu[1,#], Xiaolong Feng[1,#], Yu Pan[2*], Vicky Hasse[1], Honghui Wang[1], Bin He[1,*] & Claudia Felser[1,*]

[1]Max Planck Institute for Chemical Physics of Solids, Nöthnitzer Str. 40, Dresden 01187, Germany.

[2]College of Materials Science and Engineering and Center of Quantum Materials & Devices, Chongqing University, Chongqing 400044, China.

[#]These authors contributed equally: Haihua Hu, Xiaolong Feng.

[*]email: yupan2024@cqu.edu.cn; Bin.He@cpfs.mpg.de; Claudia.Felser@cpfs.mpg.de

Supplementary Note I. Calculation of thermopowers

In a typical two-carrier model, when $\sigma_{yx}^2 << \sigma_{xx}^2$, the S_{xx} and S_{yx} can be written as:

$$S_{xx}=\frac{S_{xx}^{e}\left(\sigma_{xx}^{e}\sigma_{xx}+\sigma_{yx}^{e}\sigma_{xx}\right)+S_{xx}^{h}\left(\sigma_{xx}^{h}\sigma_{xx}+\sigma_{yx}^{h}\sigma_{xx}\right)}{\sigma_{xx}^{2}}$$

$$S_{yx}=\frac{S_{xx}^{e}\left(\sigma_{yx}^{e}\sigma_{xx}-\sigma_{xx}^{e}\sigma_{yx}\right)+S_{xx}^{h}\left(\sigma_{yx}^{h}\sigma_{xx}-\sigma_{xx}^{h}\sigma_{yx}\right)}{\sigma_{xx}^{2}}$$

For simplicity, the Nernst thermopower S_{yx} can be expressed as:

$$S_{yx}=\frac{\sigma_{xx}^{e}\sigma_{xx}^{h}\left(\mu_{e}+\mu_{h}\right)B}{(\sigma_{xx}^{e}+\sigma_{xx}^{h})^{2}}\left(S_{xx}^{h}-S_{xx}^{e}\right)$$

where σ_{yx}^{e}, σ_{yx}^{h}, σ_{xx}^{e}, σ_{xx}^{h}, μ_{e}, μ_{h}, B, S_{xx}^{h}, and S_{xx}^{e} represent electron Hall conductivity, hole Hall conductivity, electron electrical conductivity, hole electrical conductivity, electron mobility, hole mobility, magnetic field, hole Seebeck coefficient and electron Seebeck coefficient, respectively.

In the degenerate limit, the Seebeck coefficient S_d related to the charge carrier diffusion processes can be expressed as:

$$S_{d}=\frac{8\pi^{2}m^{*}{k_{B}}^{2}T}{3eh^{2}}\left(\frac{\pi}{3n}\right)^{2/3}$$

where m^*, k_B, T, e, h, and n are effective mass, Boltzmann constant, temperature, elementary charge, Planck constant, and carrier concentration, respectively.

Hence, the Seebeck coefficient S_p related to the phonons can be written as:

$$S_p = S_{xx} - S_d$$

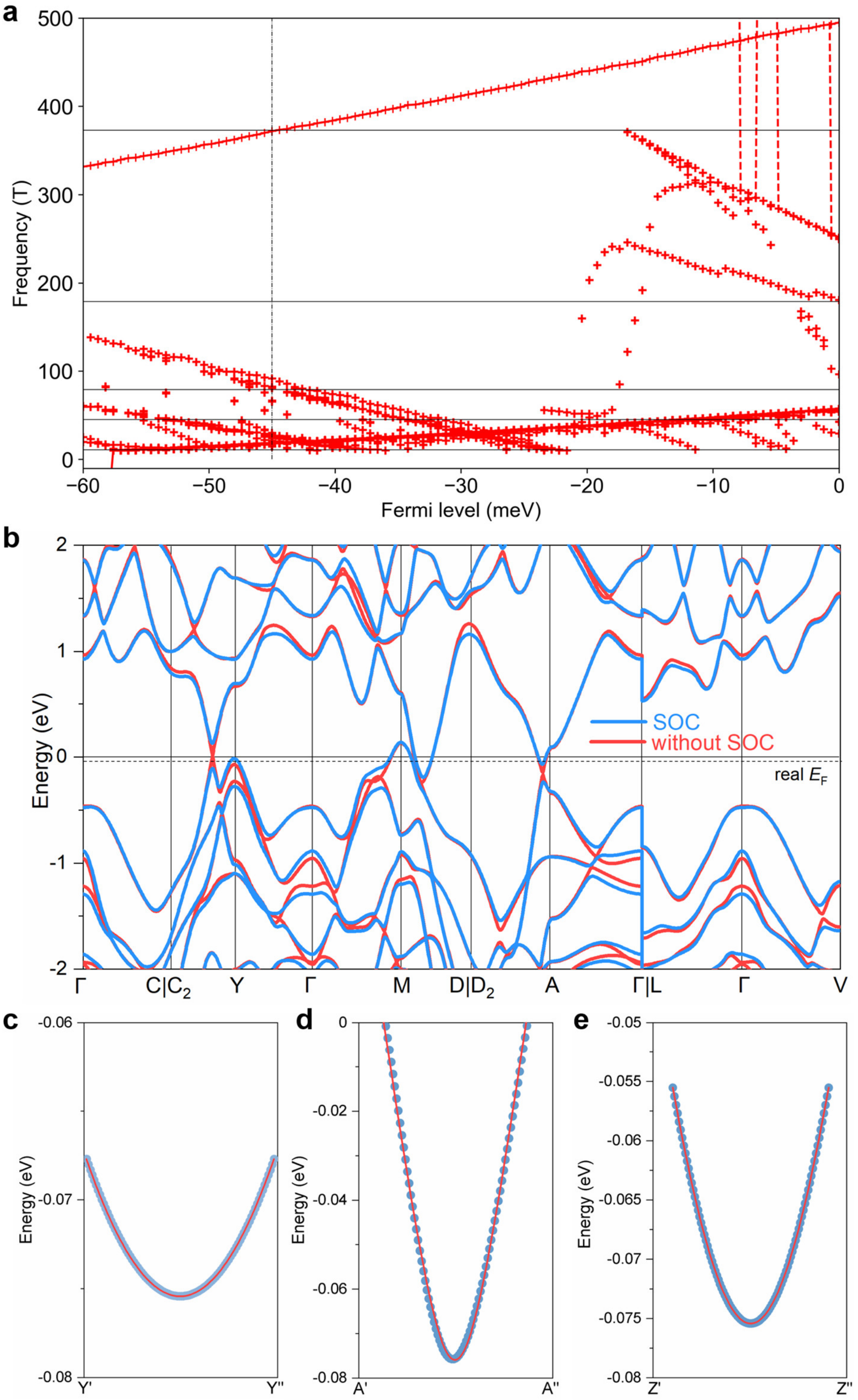

Supplementary Figure 1. DFT calculation. a. Calculated Fermi level dependent frequency. **b**. Band structures of $TaAs_2$ with and without spin-orbit coupling, respectively. **c-e**. Fitting curves of the massive Dirac band.

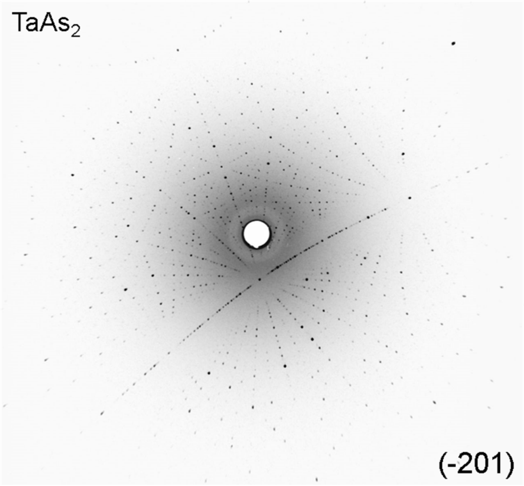

Supplementary Figure 2. single crystallinity. Laue pattern of $TaAs_2$ single crystal.

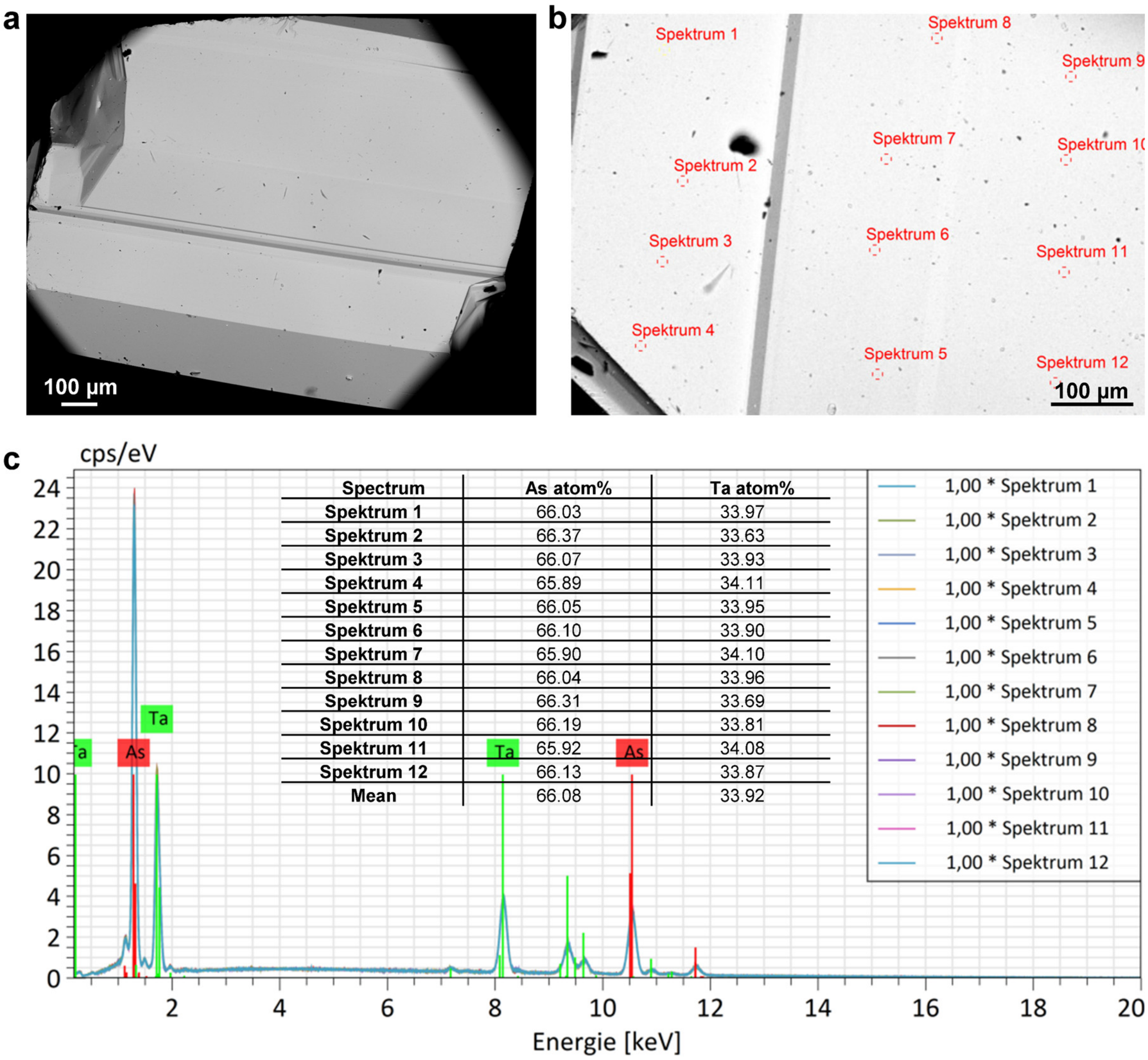

Spectrum	As atom%	Ta atom%
Spektrum 1	66.03	33.97
Spektrum 2	66.37	33.63
Spektrum 3	66.07	33.93
Spektrum 4	65.89	34.11
Spektrum 5	66.05	33.95
Spektrum 6	66.10	33.90
Spektrum 7	65.90	34.10
Spektrum 8	66.04	33.96
Spektrum 9	66.31	33.69
Spektrum 10	66.19	33.81
Spektrum 11	65.92	34.08
Spektrum 12	66.13	33.87
Mean	66.08	33.92

Supplementary Figure 3. Microstructure and composition. a Backscattered electrons image of $TaAs_2$ single crystal. **b** Measured areas of energy-dispersive X-ray spectroscopy. **c** Corresponding energy-dispersive X-ray spectroscopy and chemical composition. The absence of impurities and the Ta:As elemental ratio being close to 1:2 suggest that the sample is pure $TaAs_2$.

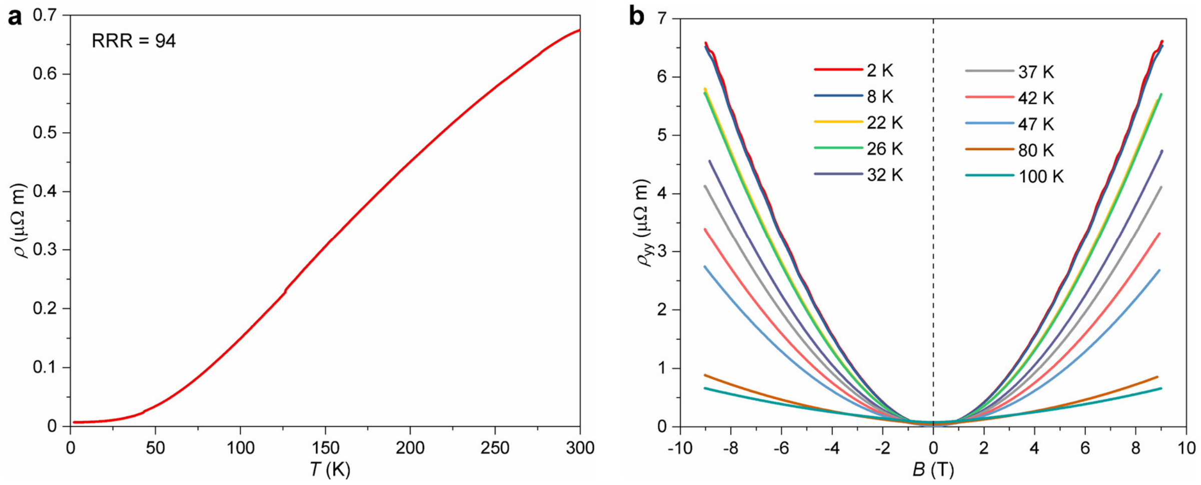

Supplementary Figure 4. Resistivity and magnetoresistance. a Temperature dependence of longitudinal resistivity in zero field. The residual resistivity ratio (RRR) value is 94. **b** Magnetic field dependence of the longitudinal resistivity ρ_{yy}.

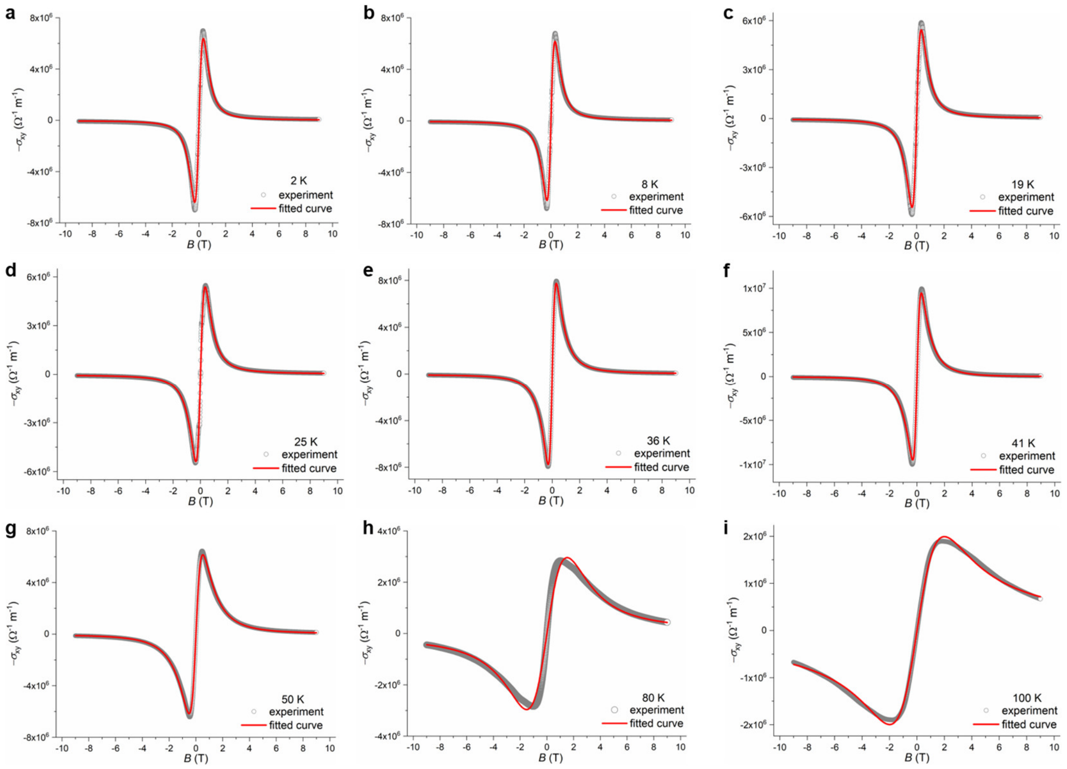

Supplementary Figure 5. Hall conductivity. Experimental values and fitting curves of the Hall conductivity are shown at various temperatures: **a** 2 K, **b** 8 K, **c** 19 K, **d** 25 K, **e** 36 K, **f** 41 K, **g** 50 K, **h** 80 K, and **i** 100 K.

By fitting the Hall conductivity σ_{xy}, the Hall charge-carrier concentration and mobility can be resolved in a system with two types of charge carrier.

$$\sigma_{xy} = \frac{\rho_{yx}}{\rho_{xx}^2 + \rho_{yx}^2} = \left[\frac{-n_e \mu_e^2}{1+(\mu_e B)^2} + \frac{n_h \mu_h^2}{1+(\mu_h B)^2} \right] eB$$

where ρ_{yx} and ρ_{xx} are the Hall and longitudinal resistivity. n_e and n_h are the electron concentration and hole concentration, respectively.

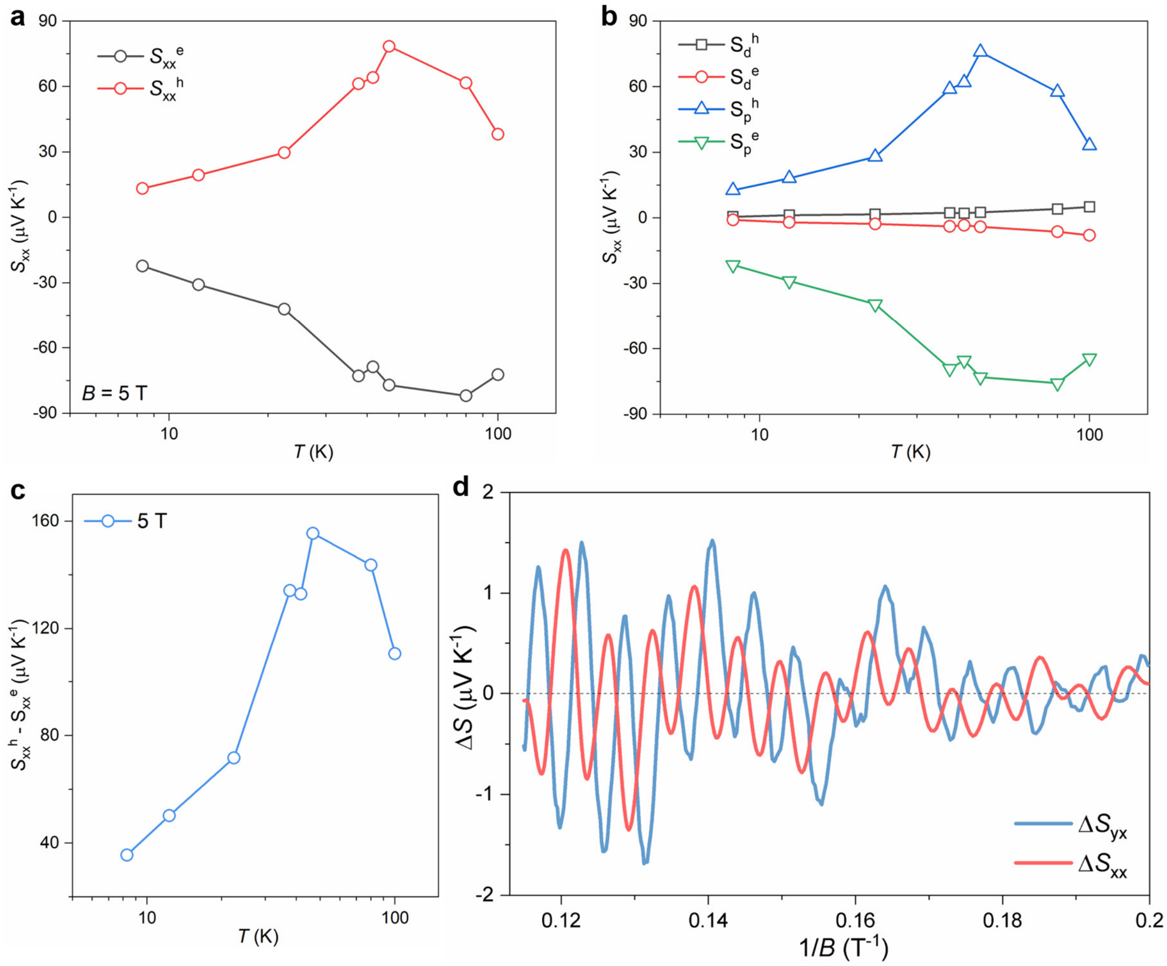

Supplementary Figure 6. Thermal transport properties. a Respective Seebeck coefficient of electrons S_{xx}^{e} and holes S_{xx}^{h} under 5 T for $TaAs_2$ derived from the two-carrier model. **b** Seebeck coefficient of electrons and holes related to the charge carrier diffusion processes (S_d^{e} and S_d^{h}) and phonons (S_p^{e} and S_p^{h}) at 5 T, respectively. **c** Temperature dependence of the difference between Seebeck coefficient of electrons and holes (S_{xx}^{h} - S_{xx}^{e}) of $TaAs_2$ under 5 T. **d** Seebeck coefficient and Nernst thermopower oscillations of $TaAs_2$ after subtracting the background as a function of $1/B$.

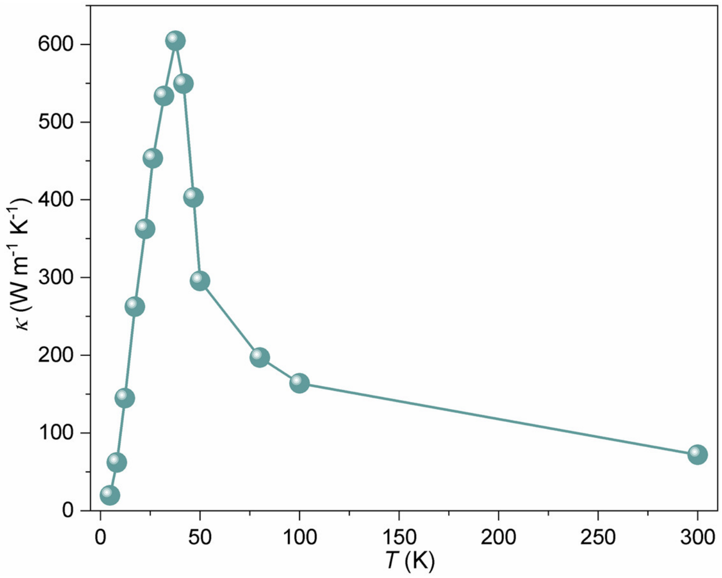

Supplementary Figure 7. Thermal conductivity. Temperature dependence of thermal conductivity.

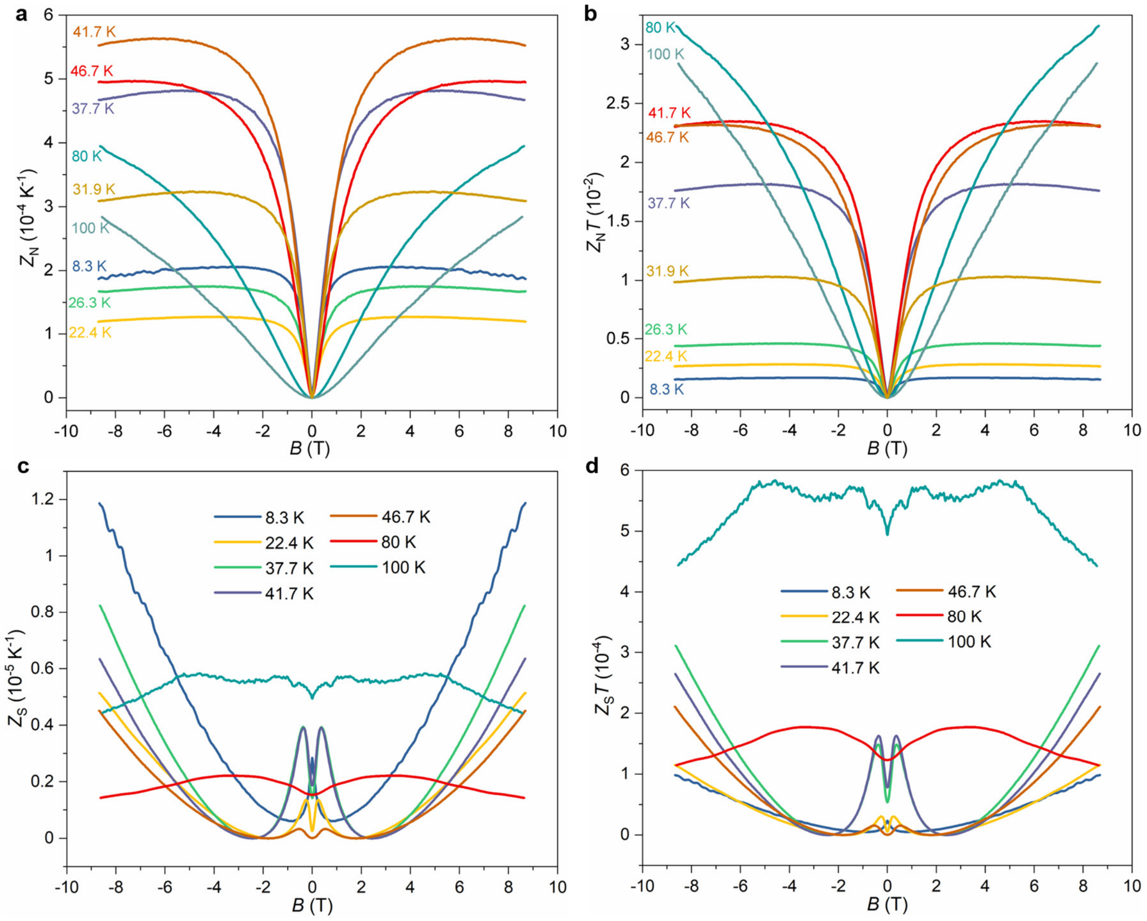

Supplementary Figure 8. Thermoelectric figure of merit. Magnetic field dependence of (a) Z_N, (b) $Z_N T$, (c) Z_S, and (d) $Z_S T$ at different temperatures.

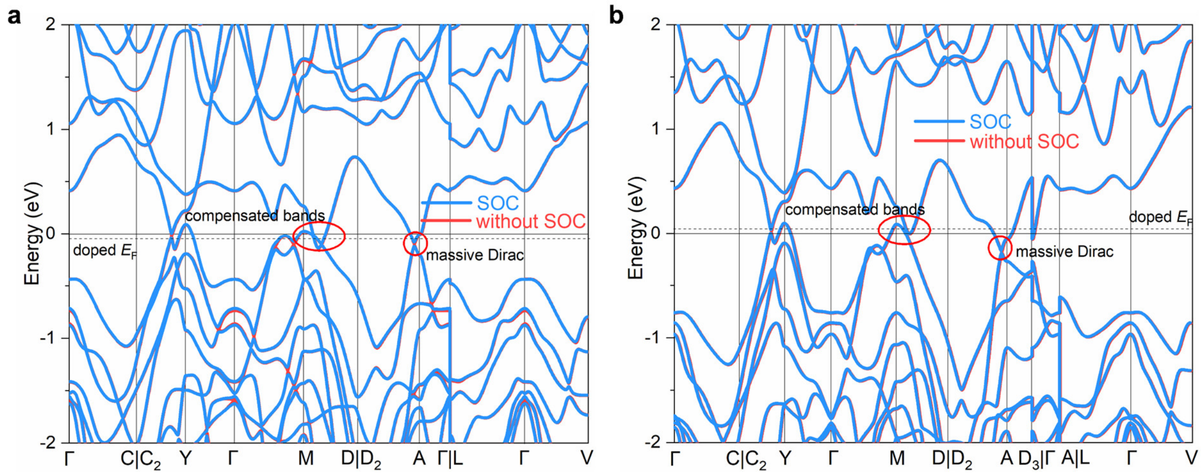

Supplementary Figure 9. Band structures. Electronic band structures of VAs_2 (a) and VP_2 (b) without and with spin-orbit coupling.
